# Knowledge and attitude on sexually transmitted infections and contraceptive use among university students in Bhutan

**DOI:** 10.1371/journal.pone.0272507

**Published:** 2022-08-03

**Authors:** Thinley Dorji, Karma Wangmo, Dendup Tshering, Ugyen Tashi, Kinley Wangdi

**Affiliations:** 1 Kanglung Hospital, Kanglung, Trashigang, Bhutan; 2 Regional Livestock Development Centre, Kanglung, Trashigang, Bhutan; 3 Sherubtse College, Royal University of Bhutan, Kanglung, Trashigang, Bhutan; 4 Ministry of Health, Kawajangsa, Thimphu, Bhutan; 5 Department of Global Health, National Centre for Epidemiology and Population Health, College of Health and Medicine, Australian National University, Acton, Australia; Al-Jouf University College of Pharmacy, SAUDI ARABIA

## Abstract

**Objectives:**

The unmet needs of contraception can lead to unintended pregnancy and transmission of sexually transmitted infections (STI). Therefore, this study aimed to evaluate the contraception use, knowledge, and attitude on STI among students under Royan University of Bhutan (RUB).

**Methods:**

This was a cross-sectional study using an online questionnaire. The questionnaire was developed in Google forms and the link was shared through the college WeChat groups. The questionnaire consisted of four parts on socio-demographic, sexual behaviour and contraceptive use, knowledge, and attitude on STIs. All the students under RUB were invited to participate voluntarily in this study. The socio-demography was presented in frequency and proportion.

**Result:**

A total of 1,283 students participated in this survey and 55.0% (701) were females. Of this, 29.4% (377) were sexually active and 94.4% reported using modern contraception. Commonly used contraceptives were: condoms (83.8%, 316) and emergency contraceptives (20.6%, 78), respectively. The mean knowledge and attitude scores on STI were 9.94 (range 2–20) and 12 (range 2–14), respectively. Good knowledge and attitude on STI were reported in 53.2% (683) and 70.1% (899) of participants.

**Conclusion:**

Students reported average knowledge and a good attitude towards STI. Contraceptive use among university students was low. There is a need to strengthen health education on STIs in schools and universities. All forms of contraceptives especially condoms should be made easily available to sexually active people.

## Introduction

The right to informed modern contraceptives is a basic human right. However, of the 1.1 billion women in the reproductive age groups who require contraception, 270 million women have unmet needs for contraception [1]. This tends to be more common among adolescent women compared to adult women [2]. An estimated 23 million adolescent girls in low and middle-income (LMI) countries do not have access to modern contraceptives [3]. The physical, biological, and hormonal changes in adolescents often drive them to engage in risky sexual acts [4]. This is further compounded by the use of alcohol and drugs among adolescents which leads to increased sexual activity [5] and indulge in unprotected sexual intercourse [6] making them prone to unintended pregnancies and sexually transmitted infections (STI).

Globally, STI causes significant morbidity and mortality especially in LMI countries [7]. More than one million cases of STI are transmitted every day, with 499 million cases comprising of gonorrhea, syphilis, chlamydia and trichomonas [7]. Untreated STI can lead to infertility, pelvic inflammatory disease, ectopic pregnancy and cervical cancer [8]. Moreover, presence of non-HIV STI increases the risk of HIV [9].

In Bhutan, STI continues to be a public health problem. The current management of STI is based on syndromic management as recommended by World Health Organization (WHO) [10]. Therefore, all patients presenting with symptoms suggestive of STI are offered counselling and tested for hepatitis, HIV and syphilis. Partner tracing is also encouraged to prevent reinfection and further spread of infection. Moreover, all pregnant women attending antenatal care undergo STI counselling and testing. In Bhutan, condoms are freely available to the public at all levels of health facilities as barrier contraceptives. In addition, condom vending machines have been installed in strategic points such as hotels to increase the uptake.

Currently, there are few studies on STIs and contraceptive use in Bhutan. A survey in 2010 in the capital city of Bhutan reported symptoms of STI in 20% and 29% of males and females, respectively [11]. This could be due to the practice of casual unprotected sex among college students [12]. The knowledge on STI and contraceptive use among college students in Bhutan have not been studied. However, knowledge of STI and contraceptive use among the young generation are important in planning preventive and education strategies [13]. Therefore, this study would identify the gaps in knowledge on STI and contraceptive use and barriers in this group to devise education and prevention strategies. So, the aim of this study were to evaluate the knowledge and attitude towards STI and contraceptive use among college students in Bhutan.

## Materials and methods

### Study design and setting

This was a cross-sectional study using a structured pre-tested questionnaire. This study was conducted in the Kingdom of Bhutan. The country is situated in the Eastern Himalayas located between China in the north, and India in the south, west and east. Bhutan has an area of 38,394 km^2^ and is administratively divided into 20 districts or *dzongkhags*.

### Study population

The study population included the students currently enrolled in colleges under the Royal University of Bhutan. These colleges are located in different parts of Bhutan. We invited all the students to participate in this online study.

### Data collection

The data was collected using a validated questionnaire adapted from a similar study [14]. The questionnaire was pre-tested among 20 students of Sherubtse College to check for any inconsistencies and these students were asked not to participate in the final survey. The questionnaire was developed in Google forms and the link was shared through the college WeChat groups. The first page of the online survey provided a brief introduction to study background, aims, declaration of confidentiality, and details of lead researchers for further questions about the study. The questionnaire became available only upon the agreement to participate in the study. The self-report questionnaire took, on average ~5–10 minutes. The data was collected over three months from 1 April to 30 June 2021.

### Measures

The questionnaire consisted of four parts: (i) socio-demographic, (ii) sexual behaviour and contraceptive use, (iii) knowledge and (iv) attitude on STI. Socio-demographic questions consisted of closed-ended questions on age, gender, year of study, residential status (hostel, self-catering, day scholars), marital status, education level of parents and occupation of parents. The questions on sexual behaviour and contraceptive use included: history of sexual activity, age at first sexual activity, contraceptive use, numbers of sexual partners, history of STI and testing, sexual activity under alcohol and drug use, adequacy of sexual health information and availability of contraceptives.

The knowledge domain consisted of nine questions with multiple correct answers. The questions were designed to test knowledge on the identification of STI, transmission, symptoms and risk factors. The questions with correct answers were given a score of “1” and “0” for wrong answers or don’t know. The total knowledge score for all the correct answers were 23 points. The attitude was measured through 14 questions and the answers were recorded on a 4-point Likert scale of strongly agree, agree, disagree and strongly disagree. A score of “1” was given for those with a good attitude and “0” for a poor attitude. Using mean score as the cutoff, the participants who scored above mean were considered as having good knowledge or good attitude. Those participants who scored less than or equal to mean were considered as having poor knowledge or poor attitude.

### Data analysis

The data were downloaded from Google Form into Microsoft Excel (Microsoft Cooperation) for cleaning and management. The data were analyzed using Stata version 13 (Stata Corporation, College Station, TX, USA). The socio-demography was presented in frequency and proportions. A Chi-square test was used to test for any differences in the different groups. *P*-value <0.05 were considered statistically significant.

### Ethical clearance

The ethical approval was provided by the Ethical Unit of Sherubtse College under the Royal University of Bhutan (No.15 (3)-SC/DRIL/2021/51). Since the survey was low risk and voluntary, the informed consent was waivered for all including minors by the ethics committee.

## Results

### Socio-demographic

A total of 1,283 students under the Royal University of Bhutan participated in this survey. The mean age of the respondents was 21.7 years (range: 17–36) with the majority of them (63.0%, n = 809/1283) in the age group of 21–24 years (**Table 1**). Fifty-five percent (n = 701/1283) were female students and 15% (n = 194/1283) of the participants were either married or living together.

**Table 1:** Sociodemographic characteristics of the participants.

Variables	Female (701)		Male (582)		Total (1,283)		*p*-value
	Number	%	Number	%	Number	%	
**Age (years)**							
17–20	242	34.5	148	25.4	390	30.4	0.001
21–24	423	60.3	386	66.3	809	63.1	
>24	36	5.1	48	8.3	84	6.5	
**Marital status**							
Single	609	86.9	480	82.5	1,089	84.9	0.028
Married*	92	13.1	102	17.5	194	15.1	
**Residence**							
Hostel (mess)	356	50.8	283	48.6	639	49.8	0.103
Self-catering	258	36.8	201	34.5	459	35.8	
Day scholar	29	4.1	40	6.9	69	5.4	
Parents/relatives	58	8.3	58	10	116	9	
**Parent’s education**							
NFE	349	49.8	335	57.6	684	53.3	0.018
<Bachelor	229	32.7	155	26.6	384	29.9	
≥Bachelor	123	17.6	92	15.8	170	17	
**Parent’s occupation**							
Farmer	357	50.9	367	63.1	724	56.4	<0.001
Government job	219	31.2	145	24.9	364	28.4	
Private/business	120	17.1	63	10.8	183	14.3	
Monk	5	0.7	7	1.2	12	0.9	

* Married or living together; NFE- non-formal education

### Sexual risk behaviour

A total of 377 (29.4%) students reported being sexually active. Around 4.1% (n = 53/1283) of participants reported having sexual intercourse under the age of <18 years. The overall mean age of sexual initiation was 19.7±2.2 (females mean age 19.4±2.3 vs 20.2± 1.9 for males). However, significant proportion of males (40.4%, n = 235) were more sexually active compared to females (20.3%, n = 142) (*p* <0.001) (**Table 2**). The major proportion of sexually active (83.8%, n = 316/377) had single partners while 16.1% (n = 61/377) had multiple sexual partners. Significant proportions of males reported having had sexual activity under the influence of alcohol (*p* = 0.015) and drugs (*p* = 0.003) compared to females. More females reported being adequately informed on sexual health compared to males (86% versus 73%, *p* = 0.003).

**Table 2:** Contraceptive use and risk behaviour of the university students of Bhutan.

Variable	Female (%)	Male (%)	Total (%)	*p*-value
**Ever had penetrative sex?**				
No	559 (79.7)	347 (59.6)	906 (59.6)	<0.001
Yes	142 (20.3)	235 (40.4)	377 (29.4)	
**Age at first sex (n = 372)**				
< 18 years	8 (5.6)	45 (19.6)	53 (14.3)	<0.001
≥18 years	134 (94.4)	185 (80.4)	319 (85.7)	
**Use some form of modern contraception**				
No	7 (4.9)	14 (6)	21 (5.6)	0.849
Yes	135 (95.1)	221 (94)	356 (94.4)	
**Sexual partners**				
No partner	559 (79.7)	347 (59.6)	906 (70.6)	<0.001
Single partner	131 (18.7)	185 (31.8)	316 (24.6)	
Multiple partner	11 (1.6)	50 (8.6)	61 (4.8)	
**Tested for STIs**				
Yes	8 (5.6)	26 (11.1)	34 (9)	0.075
No/Don’t know	134 (94.4)	209 (88.9)	343 (91)	
**Treated for STIs**				
Yes	3 (2.1)	12 (5.1)	15 (4)	0.15
No	139 (97.9)	223 (94.9)	362 (96)	
**Had sex under influence of alcohol?**				
Yes	36 (25.4)	88 (37.5)	124 (32.9)	0.015
No	106 (74.7)	147 (62.6)	253 (67.1)	
**Had sex under influence of drugs/substances?**				
Yes	2 (1.4)	21 (8.9)	23 (6.1)	0.003
No	140 (98.6)	214 (91.1)	354 (93.9)	
**Adequately informed on sexual health?**				
Yes	122 (85.9)	171 (72.8)	293 (77.7)	0.003
No/Don’t know	20 (14.1)	64 (27.2)	84 (22.3)	
**Discussed on contraception with health workers**				
Yes	32 (22.5)	85 (36.2)	117 (31)	0.006
No	110 (77.5)	150 (63.8)	260 (69)	
**Contraceptives available to you**				
Yes	93 (65.5)	150 (63.8)	243 (64.5)	0.744
No	49 (34.5)	85 (36.2)	134 (35.5)	

STIs- sexually transmitted infections

### Contraceptive use

**Fig 1:**
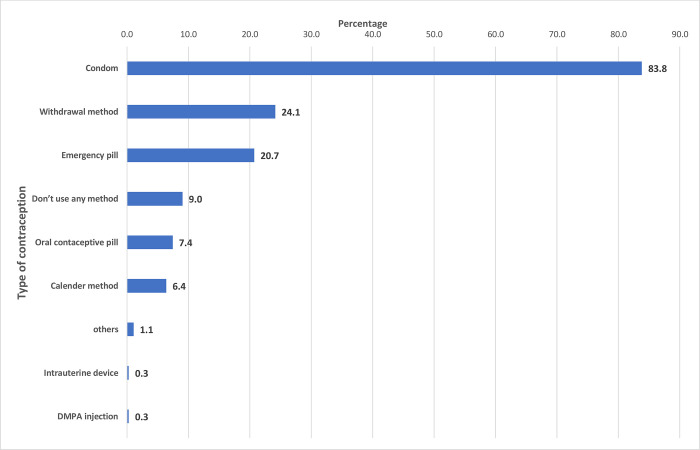
Choice of contraception among the university students of Bhutan.

**Fig 2:**
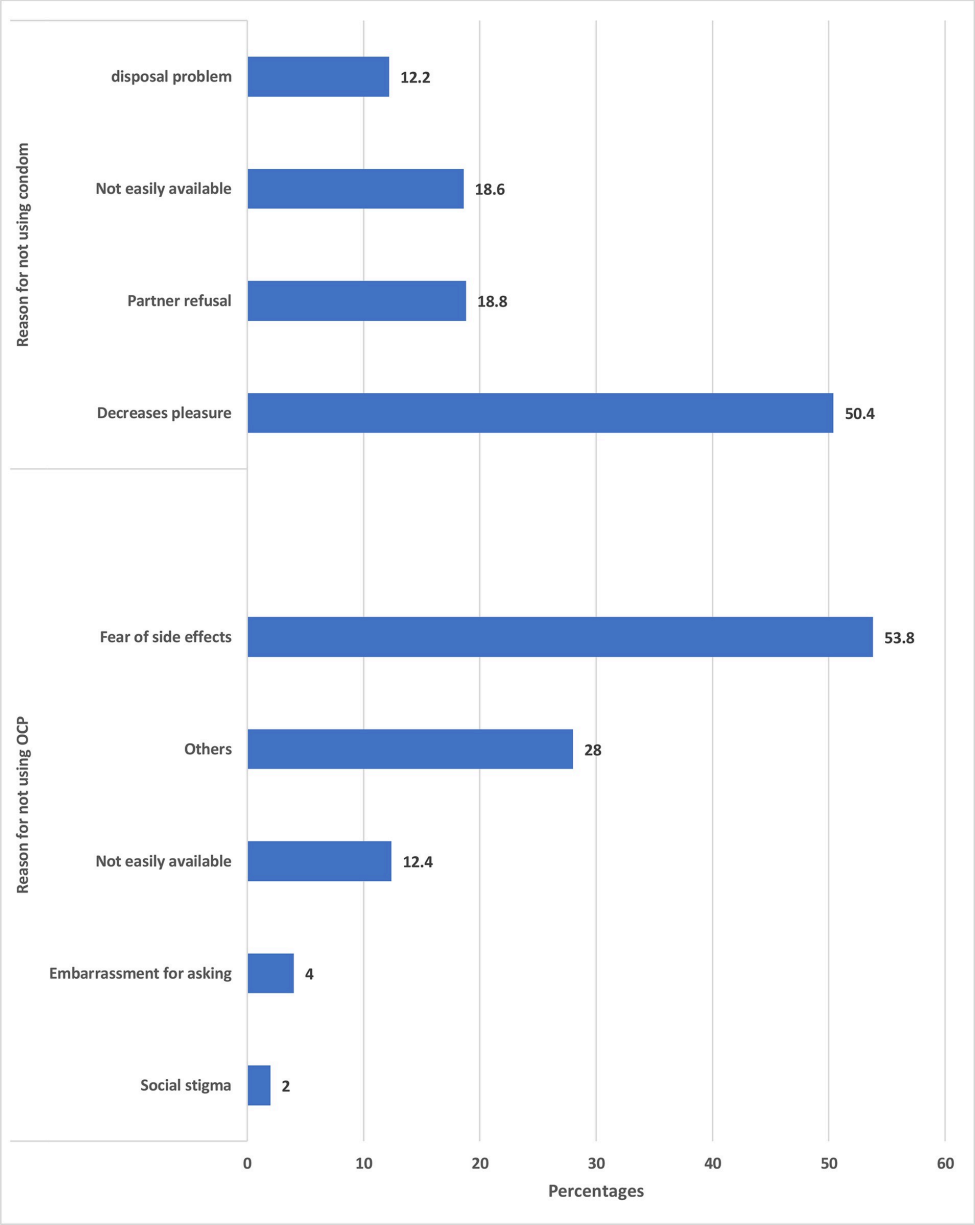
Reason for non-use of contraception among the university students of Bhutan.

Among those who were sexually active, only 94.4% (n = 356/377) reported using some form of contraception. Condom (83.8%, n = 316/) was the most commonly used contraceptive followed by withdrawal method (24.1%, n = 91/377) and emergency contraception (20.7%, n = 78/377), respectively (**Fig 1**). The reasons cited for not using a condom were decreased pleasure (50.4%, n = 190/377), partner refusal (18.8%, n = 71/377) and unavailability (18.6%, n = 70/377) at the time of sex. In this study, only 7.4% (n = 28/377) used oral contraceptive pills (OCP). The reasons for not using OCP were fear of side effects (54.0%, n = 203/377) or unavailability of OCP (12.5%, n = 47/377) (**Fig 2**).

### Knowledge of STI symptoms and transmission

The mean knowledge score on STI in this study was 9.94 (Range 2–20). Using mean as the cut-off, 53.2% (n = 683) of the participants had good knowledge of STI. The commonly identified STIs were: HIV/AIDS (93.8.%, n = 1203/1283), gonorrhea (71.9%, n = 922/1283), syphilis (31.1%, n = 399/1283) and hepatitis B (21.1%, n = 271/1283), respectively (**Table 3**). Eighty-seven per cent (n = 1,115/1283) of the participants correctly reported that STI was transmitted through unprotected sexual intercourse, sharing contaminated needles (53.4%, n = 685/1283) and mother to child transmission (38.7%, n = 496/1283). The correct symptoms of STI such as penile/vaginal discharge and painful urination were identified by 70.8% (n = 909/1283) of participants. More than one-third (36.2%, n = 464/1283) of participants wrongly reported that OCP can prevent STI transmission. The majority of participants (85.8%, n = 1,101/1283) correctly answered that condoms can prevent STIs. Just over half of participants mentioned that sexual intercourse under the influence of alcohol (53.9%, n = 692/1283) and drugs (55.2%, n = 708/1283) can lead to an increased risk of STI. Correct complications of STI including cervical cancer, infertility and pelvic inflammatory diseases were reported by 60.1% (n = 771/1283), 60.8% (n = 780/1283) and 50.1% (n = 643/1283) of participants, respectively.

**Table 3:** Knowledge of STI among the university students of Bhutan.

Characteristics		Frequency	%
**Which are of the following are STIs?**			
	HIV/AIDS	1203	93.8
	Hepatitis B	271	21.1
	Hepatitis C	106	8.3
	Syphilis	399	31.1
	Gonorrhoea	922	71.9
	Tuberculosis	32	2.5
	Rabies	26	2.0
	Asthma	7	0.5
**Routes of STIs transmission.**			
	Unprotected sexual intercourse	1,115	86.9
	Sharing contaminated needles	685	53.4
	Mother to child transmission	496	38.7
	Through blood	421	32.8
	Don’t know	135	10.5
	Kissing	63	4.9
	Contaminated food and water	20	1.6
**Oral contraceptive pills can prevent STIs.**			
	Yes	464	36.2
	No	521	40.6
	Don’t know	298	23.2
**Condom can prevent STIs?**			
	Yes	1,101	85.8
	No	36	2.8
	Don’t know	146	11.4
**Having multiple partners can increase risk of STIs?**			
	Yes	1178	91.8
	No	16	1.3
	Don’t know	89	6.9
**Sexual intercourse under the influence of alcohol can increase the risks of STIs.**			
	Yes	692	53.9
	No	132	10.3
	Don’t know	459	35.8
**Sexual intercourse under the influence of drugs can increase the risks of STI**			
	Yes	708	55.2
	No	124	9.7
	Don’t know	451	35.1
**Symptoms of STIs (multiple responses)**			
	Vaginal/penile discharge	909	70.8
	Swollen gland with fever	394	30.7
	Painful urination	937	73.0
	Genital/anal ulcer	466	36.3
	Genital warts	399	31.1
**Complications of STIs (multiple responses)**			
	Infertility	780	60.8
	Cervical cancer	771	60.1
	Pelvic inflammatory disease	643	50.1
	Ectopic pregnancy	348	27.1
	Tuberculosis	66	5.1
	Diabetes mellitus	53	4.1

STIs- sexually transmitted infections; HIV/AIDS-Human immunodeficiency virus/acquired immune-deficiency virus

### Attitude

**Fig 3:**
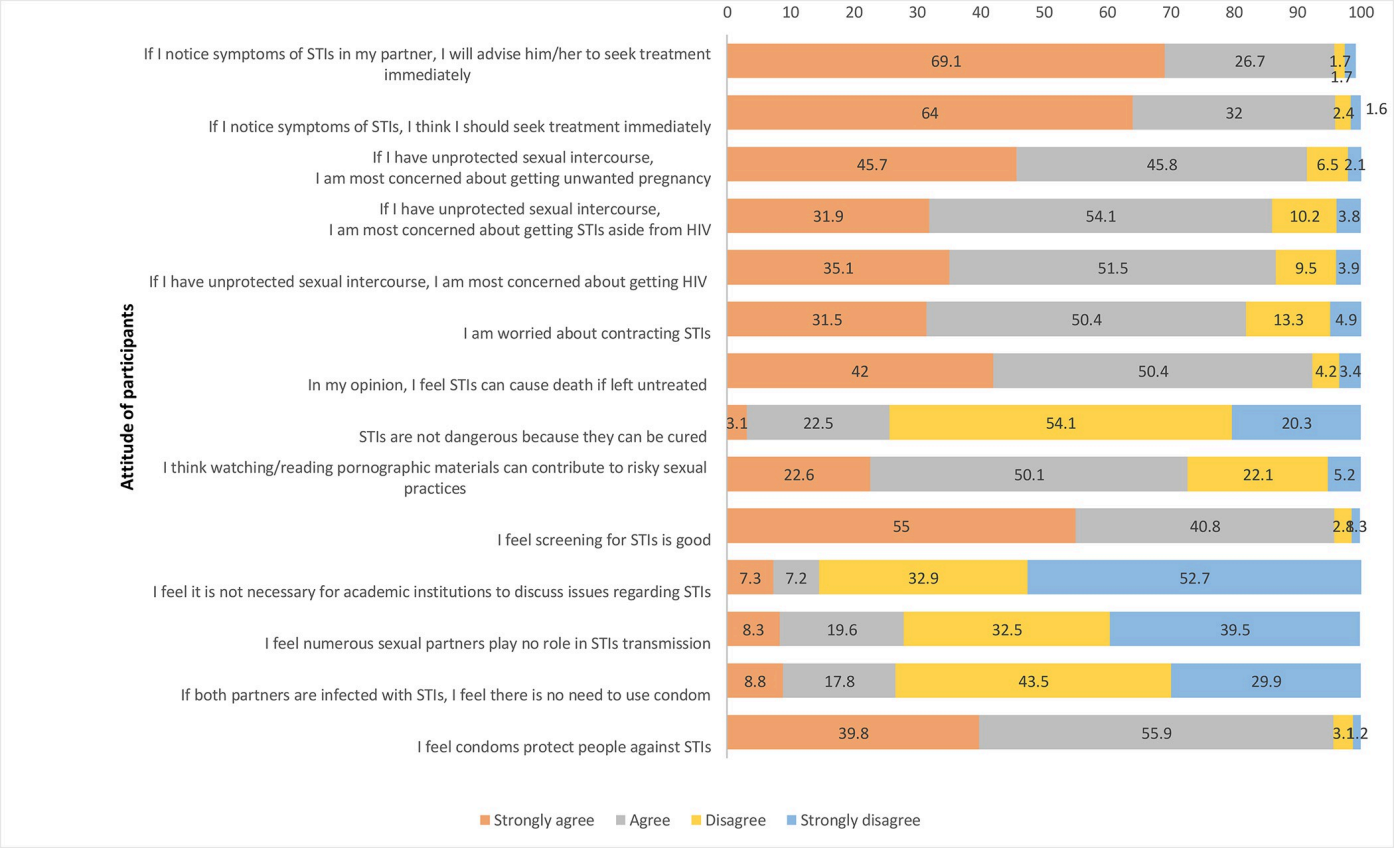
Attitude of participants towards STIs in university students of Bhutan.

The mean attitude score towards STI was 12 (Range: 2–14). Using mean as the cut-off point, 70.1% (n = 899/1283) had a good attitude towards STI. Almost 95.7% (n = 1,228/1283) agreed that the use of condoms can prevent the transmission of STI (**Fig 3**). Twenty-eight per cent (n = 359/1283) thought having multiple partners had no role in STI transmission and 26.5% (n = 341/1283) mentioned that there was no need to use condom if both the partners had STIs. Twenty-six per cent (n = 329/1283) thought that STIs were not dangerous since they can be cured. Although 91.4% (n = 1,173/1283) were concerned about unprotected sexual intercourse leading to unintended pregnancy, only 86% (n = 1,103/1283) were concerned about getting STI. Ninety-six per cent (n = 1,230/1283) thought that screening for STI is good and the same proportion reported that they would immediately seek treatment if they had symptoms of STI.

## Discussion

This study explored the contraceptive use, knowledge and attitude towards STI among university students in Bhutan. Among the sexually active participants, the use of modern contraception was high. The condom was the most commonly used contraceptive. One-third reported having had sexual activity under the influence of alcohol. The participants showed poor knowledge and good attitude towards STI. HIV was the most commonly identified STI by the respondents. Unprotected sex was identified as a risk factor for STIs.

The proportion of sexually active students in our study is much lower than a previous study on sexual health done among university students in Bhutan (30% vs 50%) [12]. This difference could be attributed to sampling strategy and time of the study. Our study used convenience sampling while the study by Gurung et al., 2016 used stratified random sampling. However, a certain level of decreasing trend of sexuality among youngsters has been observed. It is attributed to increasing use of computer games, decrease in alcohol consumption, focus on their education and career [15]. Unlike in the past, students enrolling in colleges in Bhutan are of younger age groups. Therefore, age could be one of the factors for not getting sexually exposed. The proportion of sexually active groups in our study was lower than a similar study in Portugal (76.9%) [16]. The differences could be due to differences in the study context. Compared to non-Asian students, Asian students, in general, are known to have a conservative approach towards sexual behaviour [17].

The contraceptive prevalence rate among reproductive age groups in Bhutan was 65.6% in 2021 [18] in contrast to 94.4% in our study. The proper use of modern contraception among sexually active people prevents unintended pregnancy, ectopic pregnancy and STI [19]. A study in the bordering town of Bhutan showed that lower use of contraceptives led to unintended pregnancy in 20% of the women [20]. Although abortion (except for medical reasons) is illegal in Bhutan, it is a common procedure across the international border with India [21] often with unsafe methods leading to complications and death [22]. Therefore, reproductive education should be strengthened in schools and universities.

The condom was the most commonly used contraception similar to studies from India [23] and Nepal [24]. The reason for the increased use of condoms could be due to their easy accessibility. Currently, condoms are freely available across all health centres in Bhutan. Moreover, condom vending machines are installed in strategic places such as hotels across the country. However, 18% mentioned the unavailability of the condom as the reason for non-use. This calls for efforts to make condoms and other barrier contraceptives readily available. Unlike other contraception, condom prevents both unintended pregnancies as well as STIs [25] and their use needs to be reinforced. This saves the risk as well as the cost incurred in the diagnosis and treatment of STI. The misconception such as a perceived decrease in pleasure with condom use has been reported in this study and other studies [23, 26]. Other barriers including social taboo in purchasing condoms and lack of social support [27] have been documented in studies elsewhere. The main motivating factor for contraceptive use in this study was to prevent unintended pregnancy- similar to another study [28]. Future advocacy and health campaigns should highlight the use of contraceptives especially barrier contraception for the dual purpose of prevention of unintended pregnancy and STIs.

High risk behaviour including sex under the influence of alcohol was reported in 33% of participants, which was common among males. The findings are concurrent with another study from Lebanon [29]. Alcohol use has been identified as a risk factor for high-risk behaviours such as casual sex and non-use of protection [30].

The proportion of students having good knowledge of STI in our study was just above the mean knowledge score. This is much lower than a similar study in Bangladesh (79%) [31]. This difference could be attributed to different study settings and levels of sexual education. In Bhutan, the students are not taught sexual education except for a few topics on reproductive health and STI as part of biology subject. However, the majority (94%) of the respondents mentioned HIV/AIDS as STI. The good knowledge on HIV/AIDS could be attributed due to awareness and advocacy campaigns conducted by the Ministry of Health and the annual celebration of World AIDS Day. However, this has eclipsed the knowledge on identification of STIs. Studies elsewhere have also shown that there was limited information on STIs excepting HIV/AIDS [32]. Therefore, the public should be educated on the STIs, symptoms, preventions and the available treatment modalities.

The majority of respondents were aware that STIs spread through unprotected sexual intercourse. This is in concordance with a study in Lao that also showed a high proportion of respondents were aware of the route of transmission [33]. The high usage of condoms can be partly attributed to it.

Nearly one-third of the respondents thought that multiple partners had no role in the transmission of STI. Past studies reported multiple sexual partners increased the risk of STI [34]. It is unclear whether this sexual behaviour is partly associated with cultural norms where promiscuity is accepted in Bhutanese culture. Males involved in multiple sexual partners were reported in societies that permitted polygamy [35]. Therefore, it is recommended to study the underlying reasons for this sexual behaviour in Bhutan.

These findings of this study need to be interpreted in light of the following limitations. Firstly, a causal relationship cannot be established due to the cross-sectional study design. Secondly, information was collected using a self-administered online questionnaire. The honesty and the seriousness of the respondents to the questions are difficult to assess and validate. Thirdly, contraceptive uses and other sexual behaviours were likely to be over-reported as a result of social desirability. Lastly, since this study was conducted among university students, results cannot be generalized to the general population. However, this study provides relevant information for a better understanding of knowledge and sexual behavior among students having different demographic characteristics. In addition, this study will serve as baseline information for any future studies on STI amongst university students. This can be used for assessing the effectiveness of any interventions.

## Conclusion

The contraception use among the university students in Bhutan was high with the majority preferring condoms. However, the students’ overall knowledge on STI was just above 50%. Therefore, comprehensive sexual and reproductive education in schools and colleges needs to be strengthened. This would aid in making informed decisions about one’s sexual rights and prevent unintended pregnancies and STIs. Furthermore, contraception including condoms should be made available in easily accessible locations to increase the uptake. There is a paucity of information on the challenges and barriers of delivering STI education in schools and universities. Therefore, further research on the views of teachers and students’ perceptions should be undertaken to devise/develop culturally and socially appropriate curriculum.

## Supporting information

S1 Data (DTA)
